# Rosiglitazone reverses endothelial dysfunction but not remodeling of femoral artery in Zucker diabetic fatty rats

**DOI:** 10.1186/1475-2840-9-19

**Published:** 2010-05-19

**Authors:** Xiao Lu, Xiaomei Guo, Sotirios K Karathanasis, Karen M Zimmerman, Jude E Onyia, Richard G Peterson, Ghassan S Kassab

**Affiliations:** 1 Department of Biomedical Engineering, Indiana University Purdue University (IUPUI), Indianapolis, IN 46202, USA; 2 Department of Cellular and Integrative Physiology, IUPUI, Indianapolis, IN 46202, USA; 3 Department of Surgery, IUPUI, Indianapolis, IN 46202, USA; 4 Indiana Center for Vascular Biology and Medicine, IUPUI, Indianapolis, IN 46202, USA; 5 Lilly and Company, Indianapolis, IN 46204, USA; 6 PreClinOmics, Indianapolis, IN 46268, USA; 7 Current Address: AstraZeneca R&D, Molndal, Sweden

## Abstract

**Objectives:**

Endothelial dysfunction precedes atherogenesis and clinical complications in type 2 diabetes. The vascular dysfunction in Zucker diabetic fatty (ZDF) rats was evaluated at different ages along with the effect of treatment with rosiglitazone (Rosi) on endothelial function and mechanical remodeling.

**Methods:**

The Rosi treatment was given to ZDF rats for 3 weeks. The endothelium-dependent vasodilation and α-adrenoceptor-dependent vasoconstriction of femoral arteries were studied using an *ex-vivo *isovolumic myograph. The biomechanical passive property of the arteries was studied in Ca^2+^-free condition. The expressions of endothelial nitric oxide synthase (eNOS), α-adrenoceptor, matrix metalloproteinase 9 (MMP9), and elastase were evaluated.

**Results:**

Endothelium-dependent vasorelaxation of the femoral artery was blunted at low doses in ZDF rats at 11 weeks of age and attenuated at all doses in ZDF rats at 19 weeks of age. The expression of eNOS was consistent with the endothelium-dependent vasorelaxation. The α-adrenoceptor was activated and the mechanical elastic modulus was increased in ZDF rats at 19 weeks of age. The expressions of α-adrenoceptor, MMP9, and elastase were up regulated in ZDF rats at 19 weeks of age. Rosi treatment for 3 weeks restored endothelium-dependent vasorelaxation and the expression of eNOS and the adrenoceptor activation at the doses below 10^-6 ^mole/L in ZDF rats at 19 weeks of age. Rosi treatment for 3 weeks did not, however, improve the mechanical properties of blood vessel, the expressions of α-adrenoceptor, MMP9, and elastase in ZDF rats.

**Conclusion:**

The endothelial dysfunction and mechanical remodeling are observed as early as 19 weeks of age in ZDF rat. Rosi treatment for 3 weeks improves endothelial function but not mechanical properties.

## Introduction

Cardiovascular disease (CVD) is the major cause of mortality and morbidity in people with type 2 diabetes mellitus (T2DM) and macrovascular disease is the leading cause of mortality in T2DM [1-7]. Endothelial dysfunction is a significant biomarker of early stage of CVD, which can be detected functionally as changes in vasomotor responses, cell proliferation, platelet adhesion/aggregation, vascular permeability, or leucocyte/endothelial interaction [6,8-10]. T2DM and its companion complications (atheroscleropathy) are associated with multiple metabolic toxicities and chronic injurious stimuli that result in vascular remodeling, including fibrosis, structural derangement, tissue or organ dysfunction, and ultimate failure as a result of loss of form (structure) and function [1,4,11]. Endothelial dysfunction is considered as one of the instigators of atherosclerosis during the development of T2DM and the major cause of vascular remodeling triggered by increase of growth factor release ranging from platelets to leucocytes which trigger the pathway of reactive oxygen species (ROS), and imbalance of matrix metalloproteinase (MMPs) and the collagens within vascular extracellular matrix (ECM) [1,10,11]. The changes in biomechanical properties have been documented in diabetes which reflect the micro-structural remodeling (elastin and collagen) of the vessel wall [12-17]. The endothelial dysfunction in T2DM can be improved with drug treatment through renin-angiotensin system blockers, β-blockers, Calcium antagonists, and others [7]. The drugs for lipid management have been shown to reduce macrovascular disease and improve endothelial function [3]. Both endothelial and vascular smooth muscle cells are involved in the remodeling of vessel wall in diabetes [11,16,18].

It is widely reported that vascular function is compromised in ZDF rat which is a well-established T2DM animal model [5,19,20]. Thiazolidinediones (TZD) act as agonists of the peroxisome proliferation-activated receptor γ (PPARγ), improve insulin resistance, and reduce free fatty acid (FFA) in patients with T2DM [20,21]. The TZD rosiglitazone (Rosi) enhances endothelial function and increases nitric oxide (NO) release in patients with T2DM [21,22]. Rosi has also been reported to improve endothelium-dependent vascular dysfunction in ZDF rats [5].

It is unclear if the remodeling of mechanical property of the vessel wall is reversed in T2DM animal model along with improvement of endothelial function with Rosi treatment in short term. In present study, we measured the endothelial function with a novel myograph and biomechanical properties (stress-strain relationship) of femoral artery in ZDF rats with/without Rosi treatment and their age-match groups. The biomarkers, including vascular reactivation and expression of eNOS (for endothelial functions), α-adrenoceptor (for vascular remodeling and smooth muscle hypertrophy), MMP9 (for remodeling of basement membrane of blood vessel), and elastase (for remodeling of extracellular matrices, especially elastin), were also evaluated in this study.

## Materials and methods

The animal experiments were performed in accordance with the guidelines of Institute of Laboratory Animal Research Guide, Public Health Service Policy, Animal welfare Act, and an approved PreClinOmics IACUC protocol.

### Animals and treatments

Zucker diabetic fatty (ZDF) and Zucker lean littermate rats were obtained from Charles River. ZDF and lean rats were fed with a commercial rodent chow Purina 5008 *ad libitum*. The body weight was measured weekly. The baseline glucose and triglycerides were measured in whole blood taken from the tail vein in the morning of the terminal study. The animals were randomly divided into age-matched and compound-treated groups. Age-matched groups had two subgroups: 11-weeks and 19-weeks. There were vehicle and rosiglitazone (Rosi) treated groups from 16 weeks to 19 weeks of age. The vehicle treated group was orally gavaged with 1% CMC/0.25% Ten 80 at 10 ml/kg. The compound Rosi treated group was given 3 mg/kg/day Rosi in vehicle in the morning by oral gavage as outlined in previous reports [5,20].

### Tissue preparation and isovolumic myograph

On the day of termination, the rat was weighed and blood was sampled. The animal was anesthetized with isoflurane. The left superficial femoral artery (SFA) was cannulated to measure physiologic pressure, which indicates the blood pressure in a small artery (outer diameter of SFA is about 0.4 mm). The right common femoral artery was excised quickly and placed in cold physiological saline solution (PSS in mmole/L: 119 NaCl, 4.7 KCl, 25 NaHCO_3_, 1.17 KH_2_PO_4_, 1.17 MgSO_4_, 1.6 CaCl, 5.5 Dextrose, solution gassed by 95% O_2 _5% CO_2_). The animal was euthanized by overanesthesia with isoflurane. The left common femoral artery was excised and stored at -30°C for detection of protein expression. The branches on the right common femoral artery were ligated and the vessel was cannulated with connectors. The vessel was warmed up to 37°C slowly (20 - 25 min) and equilibrated for 40 minutes at a transmural pressure of 15 mmHg and *in situ *length.

The isovolumic system consisted of a chamber with two connectors which bridge the blood vessel and thick-wall tubes [23]. A volume compensator injected PSS into the vessel to offset the permeability of the vessel wall to maintain constant volume (i.e., isovolumic myograph). The aerated (95% O_2 _5% CO_2_) PSS filled the chamber tubes before vessel cannulation. The vessel was inflated to a desired pressure, e.g., physiologic pressure. To achieve an isovolumic condition, a clamp placed on the tube between the pressurized flask and the connector was closed and the PSS in the lumen of the vessel and tubes was sealed. The vascular contraction or relaxation in response to chemical stimulation was characterized by change of intraluminal pressure while the vessel diameter was tracked simultaneously.

### Vascular functions in response to agonist/antagonist

The dose-response vasoconstriction to phenylephrine (PE, an agonist of α-adrenoceptor) was performed to evaluate the activation of α-adrenoceptor (10^-11 ^to 10^-4 ^mole/L). To evaluate endothelium-dependent dose-response relaxation to acetylcholine (ACh, 10^-12 ^to 10^-5 ^mole/L) or endothelium-independent dose-response relaxation to sodium nitroprusside (SNP, 10^-9 ^to 10^-5 ^mole/L), the vessel was pre-constricted with sub-maximal dose of PE. KCl (60 mM) was added to the bath to test vessel viability. Since diabetes and hypertension are common co-morbidities, we investigated the synergistic effect of acute over-inflation in the diabetic vessels on endothelial function. The artery was stretched to physiologic axial stretch ratio of 1.3 and inflated to a hypertensive level (170% of physiological pressure). The endothelium-dependent (ACh) and -independent (SNP) relaxations were performed according to the above protocol.

The circumferential contractile tensions were computed at every PE concentration using Eq. [1]. The percent relaxation was indicated with the ratio of change in tension at every ACh concentration or SNP concentration to the decrease in tension relative to maximal concentration. The circumferential tension was computed based on Laplace's equation:(1A)

where *T *is circumferential tension and *P *is transmural pressure. *r*_*int *_is internal radius of blood vessel related to the external radius and given by the incompressibility assumption.(1B)

where *A*_*0 *_is the cross-sectional wall area of vessel at no-load state (zero transmural pressure) and λ is axial stretch ratio.

### Passive mechanical properties

After fully relaxed in Ca^2+ ^free enviroment, the vessel segment was inflated from 0 to 160 mmHg at rate of ~4 mmHg per second and then deflated to zero. The passive pressure-diameter was recorded after 3 cycles of preconditioning. The circumferential Kirchhoff stress was computed with the following equation:(2A)

where *p *is the pressure. *r*_*int *_is the inner radius computed from Eq [1B]. *h *is wall thickness of the vessel segment (*h = r*_*ext *_*- r*_*int*_). *λ *is the circumferential stretch ratio and the midwall Green strain was computed as follow:(2B)

The elastic modulus, *E*, in the physiological state was calculated as:(2C)

where *ε*_*physio *_is the circumferential strain at physiological state.

### Expressions of eNOS, α-adrenoceptor, MMP9, and elastase

Briefly, the protein extracts (~25 μg) from arterial tissues were fractionated on 10% SDS-PAGE gel, transferred onto polyvinylidene difluoride membrane, and probed with the following primary antibodies: anti-eNOS (BD transduction laboratory), anti-α-adrenoceptor (ABCam), anti-MMP9 and anti-elastase (Santa Cruz Biotech). Blots were incubated with horseradish peroxidase-conjugated secondary antibody. The signal was detected by enhanced chemiluminescence (ECL, Amersham) and evaluated by densitometry (Sigma Scan). β-actin was used for normalization.

### Statistics

The data were presented as mean ± SD. Significant differences between two data points were determined by student *t*-test. Significant differences between the dose-dependent groups and stress-strain relationship were determined by use of Analysis Of Variance (ANOVA) between groups. A probability of p < 0.05 was considered to be indicative of a statistically significant difference.

## Results

The ages and numbers of animals in the various groups are represented in Table 1. The average blood pressure in superficial femoral artery (Table 1) were not significantly different in various groups (P > 0.05), This result indicates that the blood pressure in a smaller artery is similar to central arterial pressure which does not significantly increase in ZDF rats as previously reported [5]. The body weight increased in lean rats from 11 to 19 weeks of age but not in ZDF rats (Table 1). Although the vehicle treatment did not change the body weight, Rosi treatment increased the body weight of ZDF rats similar to previous reports [5,21]. Plasma glucose (Table 1) was significantly increased in ZDF rats (p < 0.05) in both 11 and 19 weeks of age but not in lean rats (p > 0.1). Plasma glucose in the Rosi treated ZDF rats did not decrease significantly (p > 0.05) and remained at high concentration. The concentrations of plasma triglycerides were significantly increased in ZDF rats but not statistically changed (p > 0.05) after Rosi treatment (Table 1).

**Table 1:** Effect of age and the treatment of Rosiglitazone on body weight, average arterial pressure, blood glucose, and triglycerides.

	11 weeks		19 weeks		19 weeks	
	
Determination	Lean	ZDF	Lean	ZDF	Veihcle	Rosi
Number of Animals	6	7	6	6	6	6
Age of Animal (weeks)	11.8 ± 1.2	11.3 ± 0.9	19.7 ± 1.5	19.2 ± 1.1	19.6 ± 1.5	19.3 ± 1.2
Average Pressure (mmHg)	69.2 ± 8.9	71.7 ± 9.3	72.2 ± 9.7	74.8 ± 11.6	71.9 ± 10.3	73.9 ± 11.8
Body Weight (grams)	339 ± 38	369 ± 35	382 ± 35	401 ± 37*	403 ± 29*	423 ± 33*
Glucose (mg/dl)	112.8 ± 16.1	457.8 ± 35.9*	116.3 ± 15.0	431.4 ± 30.3*	472.0 ± 46.0*	445.6 ± 48.8*
Triglyceride (mg/dl)	108.3 ± 11.9	583.4 ± 49.5*	121.8 ± 16.5	602.6 ± 59.7*	609.1 ± 79.7*	613.1 ± 46.5*

Notes: Lean: Zucker lean rats. ZDF: Zucker diabetic fatty rats. Rosi: rosiglitazone treated. * p < 0.05 compared with lean rats.

**Figure 1:**
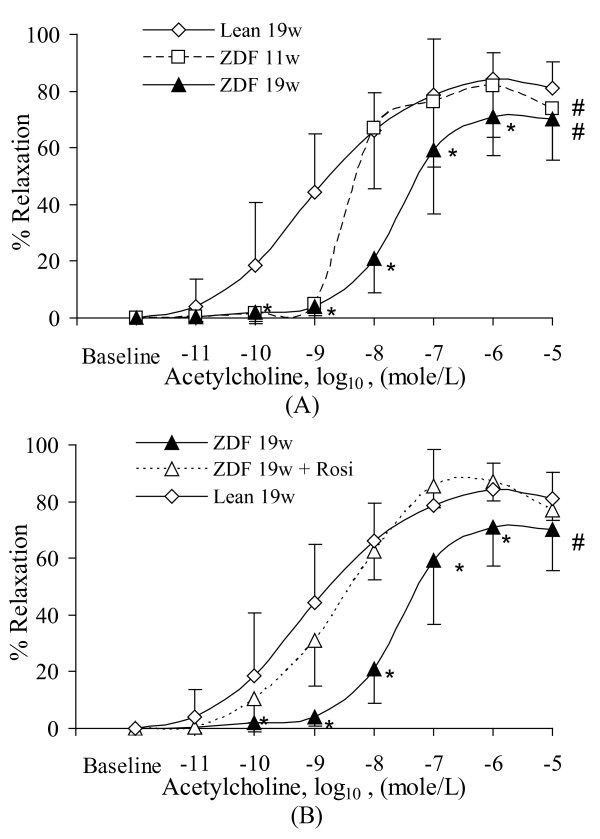
**The endothelium-dependent dose-response vasorelaxation for the various groups**. **A**: Effect of age (11 wks and 19 wks) on the vasorelaxation of ZDF rats in comparison with lean rats. **B**: Effects of Rosi on the vasorelaxation of ZDF rats in comparison with lean rats. Lean: Zucker lean rats. ZDF: ZDF rats. Rosi: rosiglitazone treatment. 11w: 11 weeks of age. 19w: 19 weeks of age. #: ANOVA analysis indicates statistical difference (p < 0.05) of the dose-dependent curve between the ZDF rats at different age and lean rats. *: Significant difference (p < 0.05) at single dose between ZDF rats at different age and lean rats.

Figure 1A shows endothelial dysfunction as early as 19 weeks of age in ZDF rats (p < 0.05). Even at 11 weeks of age, the endothelium-dependent relaxation of ZDF rats was blunted at concentrations lower than 10^-9 ^mole/L (Figure 1A) in comparison with lean rats (p < 0.05). After treatment with Rosi, endothelial function recovered to the level of lean animal (p > 0.05) despite a significantly elevated concentration of plasma glucose (Figure 1B). Endothelium-independent relaxation to SNP failed to reveal differences before and after Rosi treatment (data not shown) and served as control.

**Figure 2:**
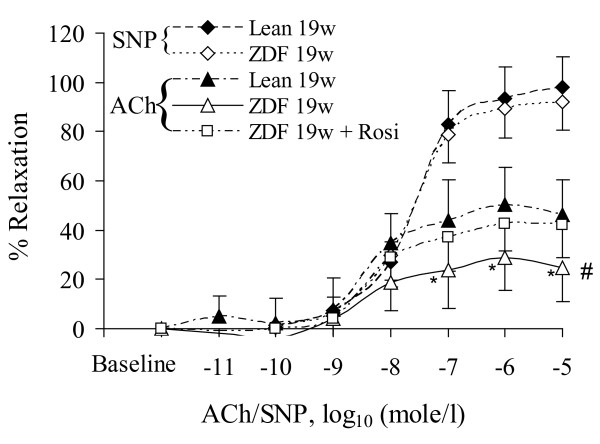
**The deleteriousness effect of acute over-inflation on the endothelium-dependent (ACh) and endothelium-independent (SNP) vasorelaxation**. Lean: Zucker lean rats. ZDF: ZDF rats. Rosi: rosiglitazone treatment. 19w: 19 weeks of age. #: ANOVA analysis indicates statistical difference (p < 0.05) of the dose-response curves of ZDF rats between SNP and ACh during acute over-inflation. *: Significant difference (p < 0.05) at the single dose between lean and ZDF rats during acute over-distension.

In Figure 2, we observed the effect of acute over-inflation (170% of physiologic pressure) in the ZDF and lean vessels on endothelial functions. The acute over-stretch significantly augmented endothelium-dependent dysfunction of artery in ZDF rats as compared to lean rats (p < 0.05). In the condition of acute over-stretch, the Rosi treatment essentially restored the endothelium-dependent relaxation of artery in ZDF rats to the level of artery in lean rats.

**Figure 3:**
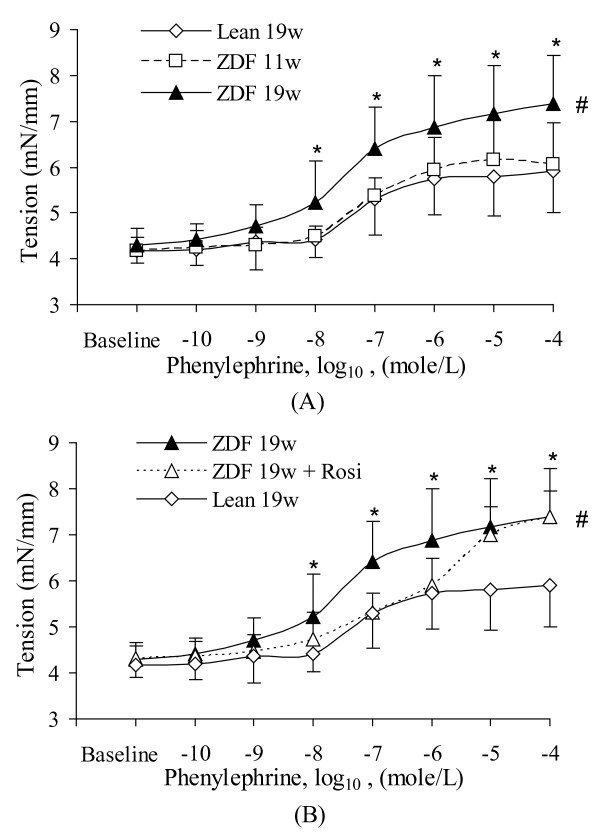
**The dose-response relation of circumferential tension for the various groups**. **A**: Effect of age (11 weeks and 19 weeks) on vasoconstriction. **B**: Effects of Rosi treatment on vasoconstriction. Lean: Zucker lean rats. ZDF: ZDF rats. Rosi: rosiglitazone treatment. 11w: 11 weeks of age. 19w: 19 weeks of age. #: ANOVA indicates significant difference (p < 0.05) of the dose-response curve between the ZDF rats at different age and lean rats. *: Statistical difference (p < 0.05) at single dose between ZDF rats at different age and lean rats.

The circumferential tension increased (p < 0.05) during PE stimulation in ZDF rats at 19 weeks of age (Figure 3A) but not at 11 weeks of age (p > 0.05). The circumferential tensions in response to 60 mM KCl were not statistically different among various ages of lean and ZDF animals (5.1 ± 1.2 kPa in lean animals at 19 weeks of age, 5.2 ± 0.9 kPa at 11 weeks of age, and 5.0 ± 1.3 kPa at 19 weeks of age). This observation implies that the adrenergic vasoconstriction is augmented in ZDF rats and similar to the observation in Zucker obese rat [24]. Rosi treatment restored the adrenergic vasoconstriction in the range of low concentration of PE (<10^-6 ^mole/L). At high concentrations of PE (1 × 10^-5 ^mole/L), the vasoconstriction was the same as without treatment (Figure 3B).

**Figure 4:**
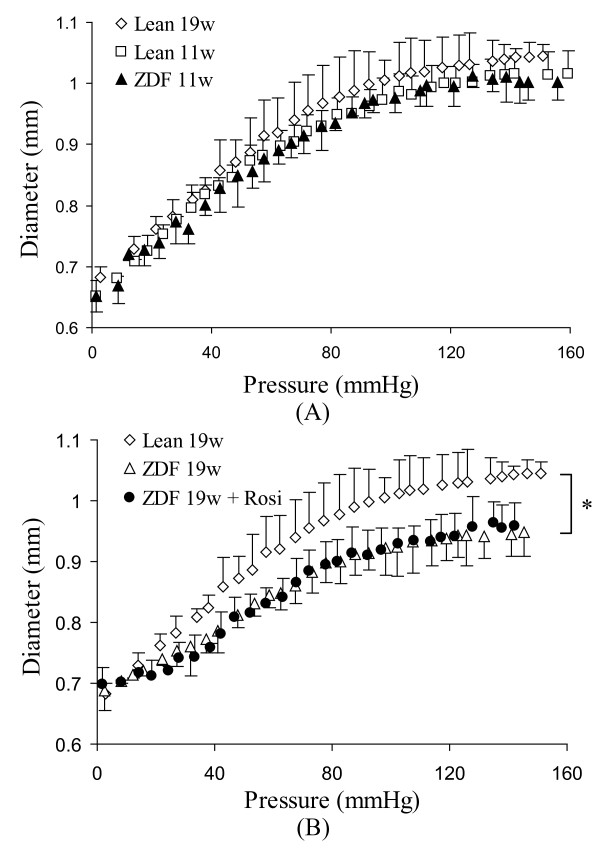
**The passive pressure-diameter relationship of the femoral artery**. The vessel segment was fully relaxed in Ca^2+ ^free PSS at 37°C. **A**: The animals in different ages: lean rats (11 weeks of age and 19 weeks of age) and ZDF rats (11 weeks of age). There is no significant difference of pressure-diameter relationship among the three groups. **B**: The animals in different groups: age-matched lean rats (19 weeks of age), ZDF rats (19 weeks of ages), and ZDF rats treated with Rosi. There is significant shift of diameter in ZDF rats at 19 weeks of age and Rosi treated ZDF rats in comparison with age-matched lean rats. Lean: Zucker lean rats. ZDF: ZDF rats. Rosi: rosiglitazone treatment. 11w: 11 weeks of age. 19w: 19 weeks of age. * p < 0.05 indicates the statistical significance between groups using ANONA analysis.

**Figure 5:**
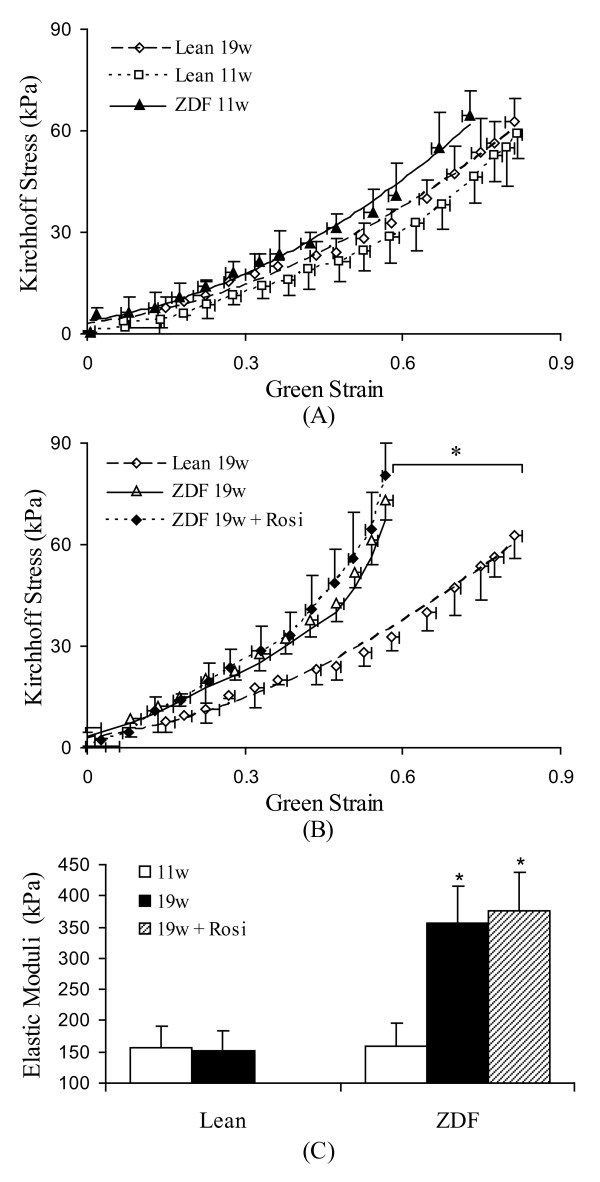
**The Kirchhoff stress- Green strain relationship of the femoral artery**. A: The animals in different ages: lean rats (11 weeks of age and 19 weeks of age) and ZDF rats (11 weeks of age). There is no significant difference of stress-strain relationship among the three groups. B: The animals in different groups: age-matched lean rats (19 weeks of age), ZDF rats (19 weeks of age), and ZDF rats treated with Rosi. In 19 weeks of age, the stress-strain curve of ZDF rats was significantly shifted to left. The Rosi treatment did not reverse the mechanical properties of the arteries. * p < 0.05 indicates the statistical significance between groups using ANONA analysis. C: The elastic moduli at the pressure near physiological state. Lean: Zucker lean rats. ZDF: ZDF rats. Rosi: rosiglitazone treatment. 11w: 11 weeks of age. 19w: 19 weeks of age. * p < 0.05 indicates the statistical significance in comparison with lean rats using student *t*-test.

The passive pressure-diameter and stress-strain relationship were presented in Figure 4 and 5. There were no significant changes of pressure-diameter and stress-strain relationship between 11 weeks and 19 weeks of age in lean rats. The vessel of ZDF rats at 11 weeks of age was not statistically stiffer in comparison with age-matched lean rats (11 weeks of age) (Figure 5A). However, the vessel of ZDF rats at 19 weeks became significantly stiffer (p < 0.05) in comparison with lean rats at 19 weeks of age (Figure 5B). The treatment of Rosi did not restore the stress-strain relationship and the elastic modulus did not change in comparison with untreated group (Figure 5C).

**Figure 6:**
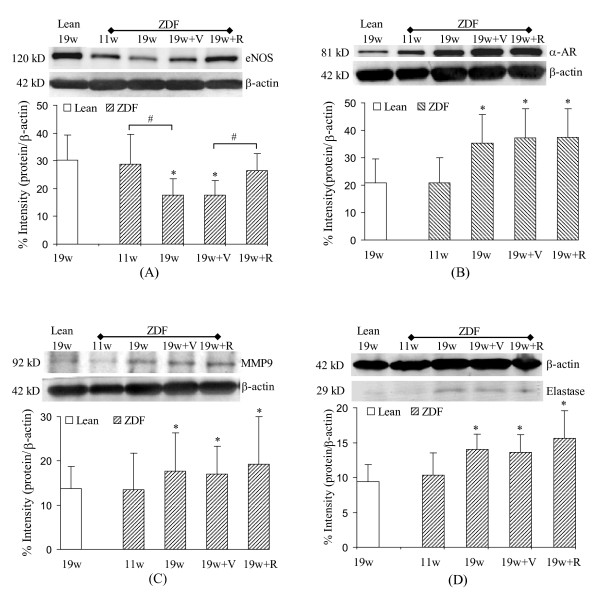
**The vascular expressions of eNOS, α-adrenoceptor (α-AR), MMP9, and elastase**. In all four sections, top panel is the bands of proteins and bottom panel is the percent intensity of the ratio of the bands to β-actin. **A**: The expression of eNOS. **B**: The expressions of α-adrenoceptor. **C**: The expressions of MMP9. **D**: The expression of elastase. Lean: Zucker lean rats. ZDF: ZDF rats. 11w: 11 weeks of age. 19w: 19 weeks of age. 19w + V: 19 weeks of age treated with vehicle. 19w + R: 19 weeks of age treated with Rosi. * p < 0.05 indicates statistical significance when compared with lean rats using student *t*-test. # p < 0.05 indicates statistical significance in comparison of the two groups using student *t*-test.

The expression of eNOS significantly down regulated in ZDF rats at 19 weeks of age but not at 11 weeks of age (Figure 6A). The expression of α-adrenoceptor up regulated in ZDF rats at 19 weeks of age but not at 11 weeks of age (Figure 6B). The increase in vasoconstriction in response to PE implies enhanced activity of α-adrenoceptor in ZDF rats at 19 weeks of age. The up regulation of MMP9 indicates remodeling of vessel wall (Figure 6C) and may correlate with endothelium function since the target of MMP9 is the basement membrane of blood vessel. We also observed up regulation of elastase in ZDF rats at 19 weeks of age (Figure 6D), where elastase is a well-known biomarker of vascular remodeling. The Rosi treatment did not reverse the up regulation of the expressions of either α-adrenoceptor, MMP9, or elastase in the vessel wall (Figure 6). The treatment of Rosi reversed the expression of eNOS, however, which is consistent with the observation on the endothelium-dependent vasorelaxation in this study.

## Discussion

In present study, the endothelium-dependent vasorelaxation in response to ACh was attenuated and the expression of eNOS down regulated in ZDF rats at 19 weeks of age in comparison to Zucker lean rats. The acute over-inflation (simulates acute hypertension) compromised the endothelium-dependent relaxation of artery in both ZDF and lean rats but more so in the ZDF rats. The passive stress-strain relationships reflect the biomechanical properties of blood vessel and a leftward shift suggests an increased elastic modulus at 19 weeks of age in ZDF rats. This is consistent with our previous observation of stiffer arteries in streptozotocin-induced diabetes [17]. The treatment for 3 weeks of Rosi improved endothelial function but did not alter the passive mechanical properties. The up regulations of α-adrenoceptor, MMP9, and elastase, provide further evidence of vascular remodeling in ZDF rats with/without Rosi treatment.

ZDF rats are inbred from a colony of obese Zucker rats with a hyperglycemic phenotype. The blood glucose in all fatty males of ZDF rat model increases more than 4-fold by 10 weeks of age when fed Purina 5008 and remains at this high level throughout their lifespan. Although between 10-13 weeks of age, the serum insulin is elevated, it declines progressively with advancing age. Between 22-42 weeks of age, serum insulin levels decline to below the levels of insulin in age-matched lean control rats due to pancreatic beta cell failure and renal injury develops [19]. In the present study, the ages of the animals were selected at 11 weeks of age at the initiation of hyperglycemia and at 19 weeks of age when diabetic complications had not significantly occurred. Endothelial dysfunction has been reported in ZDF rats at 16-24 weeks in coronary artery and over 22 weeks in aorta [5,8].

Here, we report endothelial dysfunction in significantly younger rats in the common femoral artery. Endothelial dysfunction of common femoral artery was detected in ZDF rats as early as 11 weeks in response to low concentration of ACh which may be attributed to the more physiologic loading in both inflation and axial elongation. Hypertension is very frequent among T2DM [2]. The occurrence of diabetes and hypertension are multiplicative risk factors for macro- and micro- vascular diseases and endothelial dysfunction is considered as one of the biomarkers of atherosclerosis during the development of T2DM [6]. Our results show that acute hypertension causes profound endothelial dysfunction in ZDF rat. Hence, distension of a ZDF vessel causes a confounding deleterious effect on endothelial function.

The vasoconstriction in response to PE may reflect the activation of the α-adrenoeceptor. The up regulation of α-adrenoceptor's activation and expression implies vascular hypertrophy and remodeling [25-27]. T2DM often lead to matrix deposition and glycation in blood vessel wall, particularly those proteins associated with the basement membrane, and increase in stiffness or decrease in distensibility of blood vessel [11]. The up regulation of MMP9 indicates matrix remodeling and especially the remodeling of basement membrane. The increased expression of elastase reflects degradation of elastin fibers and hence increased stiffness of the vessel wall.

Rosi treatment restored the compromised endothelium-dependent vasorelaxation in ZDF rats at the 19 weeks of age while plasma glucose and serum triglycerides remaining at high concentration consistent with previous observations [21,28,29]. It has been suggested that Rosi may prevent free fatty acid-induced endothelial dysfunction and exert anti-inflammatory and antioxidative effects directly in the vasculature [20,30-33]. In this study, we found that Rosi treatment of short time (3 weeks) blunts adrenergic vasoconstriction in <10^-6 ^mole/L of PE. Stepp and Frisbee [24] suggested that it is helpful to normalize blood pressure by blockade of the adrenergic receptor. Rosi may have beneficial effects in T2DM by partial attenuation (at low concentration) of adrenergic over-response in addition to the improvement of endothelial function. Rosi failed to restore the passive stress-strain relationship and did not have an effect on increased vascular stiffness after 3 weeks of treatment. Glycation and matrix deposit result from high glucose and the proliferation of vascular smooth muscle cells which might be elicited by activated α-adrenegic receptor. Rosi did not affect the plasma glucose and the expressions of α-adrenoceptor, MMP9, and elastase in the present study and hence did not restore the altered mechanical properties of the vessel wall. In fact, previous observations showed that Rosi increased body weight and did not improve systemic blood pressure, plasma glucose, cholesterol, triglycerides, free fatty acids, etc. [5,20,21]. The improvement of endothelial function from Rosi is not the benefit of weight loss or the reduction of glucose, triglycerides, and cholesterol. The mechanistic pathways of Rosi action requires further study.

The mechanical property of blood vessel is one of the indicators of vascular remodeling [13-17,34,35] and reflects the make up of extracellular matrices. Our results indicate that the blood vessel becomes stiffer if it is subjected to hyperglycemia for 6 weeks. The hardening of blood vessel is recognized as one of the mechanisms of diabetic hypertension and may result in vascular dysfunction. The treatment of Rosi for 3 weeks in this study failed to reverse the hardening of blood vessel wall and the associated remodeling indicated by the expression of MMP9 and elastase. Reddy, *et al *showed that treatment with ciglitazone (an agonist of PPARγ) for 8 weeks ameliorated the decrease in elastin contents [16]. The longer term effect of Rosi on mechanical property and remodeling requires future work.

In summary, the endothelial dysfunction can be observed in as early as 19 weeks of age in ZDF rat. The adrenoceptor is activated and the remodeling of extracellular matrices is implicated in diabetic complication in early age. This study shows that Rosi treatment restores the endothelial function and adrenergic vasoconstriction at low concentration of PE. The mechanical properties along with the expressions of α-adrenoceptor, MMP9, and elastase are, however, not reversed by three weeks of Rosi treatment.

## Competing interests

The authors declare that they have no competing interests.

## Authors' contributions

XL contributed to the design of the study and carried out the vascular reactive and biomechanical studies. XG carried out the molecular biological studies. SK was responsible for the overall design of the study. KZ participated in the animal studies. JO participated in the design plan. KP participated in the animal studies and physiological data collection. GK was responsible for the overall design of the study and revision of drafts. All authors read and approved the final manuscript.
